# Artery of Percheron Infarct: An Acute Diagnostic Challenge with a Spectrum of Clinical Presentations

**DOI:** 10.7759/cureus.3276

**Published:** 2018-09-10

**Authors:** Javed L Khanni, Joel A Casale, Adriana Y Koek, Patricio H Espinosa del Pozo, Patricio S Espinosa

**Affiliations:** 1 Clinical Biomedical Science, Florida Atlantic University Charles E. Schmidt College of Medicine, Boca Raton, USA; 2 Internal Medicine, Florida Atlantic University Charles E. Schmidt College of Medicine, Boca Raton, USA; 3 Neurosciences, Universidad Central Del Ecuador, Quito, ECU; 4 Neurology, Marcus Neuroscience Institute, Boca Raton Regional Hospital, Boca Raton, USA

**Keywords:** artery of percheron, stroke, thalamic infarct, paramedian thalamic infarct, bilateral thalamic infarct, thalamus, thalamic vasculature, ischemic stroke

## Abstract

The artery of Percheron (AOP) is a variant of the paramedian thalamic vasculature that supplies blood to the medial aspect of the thalamus and the rostral midbrain. The presentation of an infarct in this territory varies widely and is often characterized by nonspecific neurological deficits, with altered mental status, decreased level of consciousness, and memory impairment being among the most common. AOP infarcts are often missed on initial computed tomography (CT) scan, and additional imaging is usually not done due to low suspicion for stroke in most cases. There have been an increasing number of reports of AOP infarction, illustrating the diversity of clinical presentations and the challenge this presents to clinicians in the acute setting. Lacking the classic signs of stroke, many of these patients experience a delay in recognition and treatment, with the majority of diagnoses occurring outside the tissue plasminogen activator (tPA) window. This case highlights the unusual presentation and diagnostic difficulty of a patient with an AOP infarct, and serves as a reminder to include thalamic pathology in patients presenting with vague neurological symptoms and no obvious signs of stroke.

## Introduction

The artery of Percheron (AOP) is an anatomic variant of the blood vessels supplying the thalamus that has been observed in up to one third of human brains [1]. Occlusion of the AOP is one of the few single-artery pathologies that can affect bilateral structures. Given the range of neurological functions encompassed by the thalamus, an insult to this area may manifest clinically in a variety of ways. Ischemic strokes in more common locations, such as the middle cerebral artery, most often produce predictable focal neurological deficits and clinical syndromes. In contrast, the presentations of someone with an AOP infarct include altered mental status, coma, transient or episodic loss of consciousness, memory impairment, psychosis, aphasia, dysarthria, and oculomotor dysfunction [2-9]. When there is an acute stroke in the AOP territory, the diagnosis is often missed due to the unusual presenting symptoms and signs, as well as the fact that it may not be visualized on primary imaging. Here, we describe the case of a patient who presented with lethargy and unresponsiveness due to an AOP infarct.

## Case presentation

A 59-year-old Haitian male with a past medical history of uncontrolled diabetes mellitus was found unresponsive at work. He is a landscaper and was taking his usual lunchtime nap under a tree when his coworkers could not awaken him, prompting them to call emergency services. Upon reaching the patient, paramedics administered 0.5 mg of naloxone intravenously with no effect. Still unarousable, he was transported to the emergency department.

**Figure 1:**
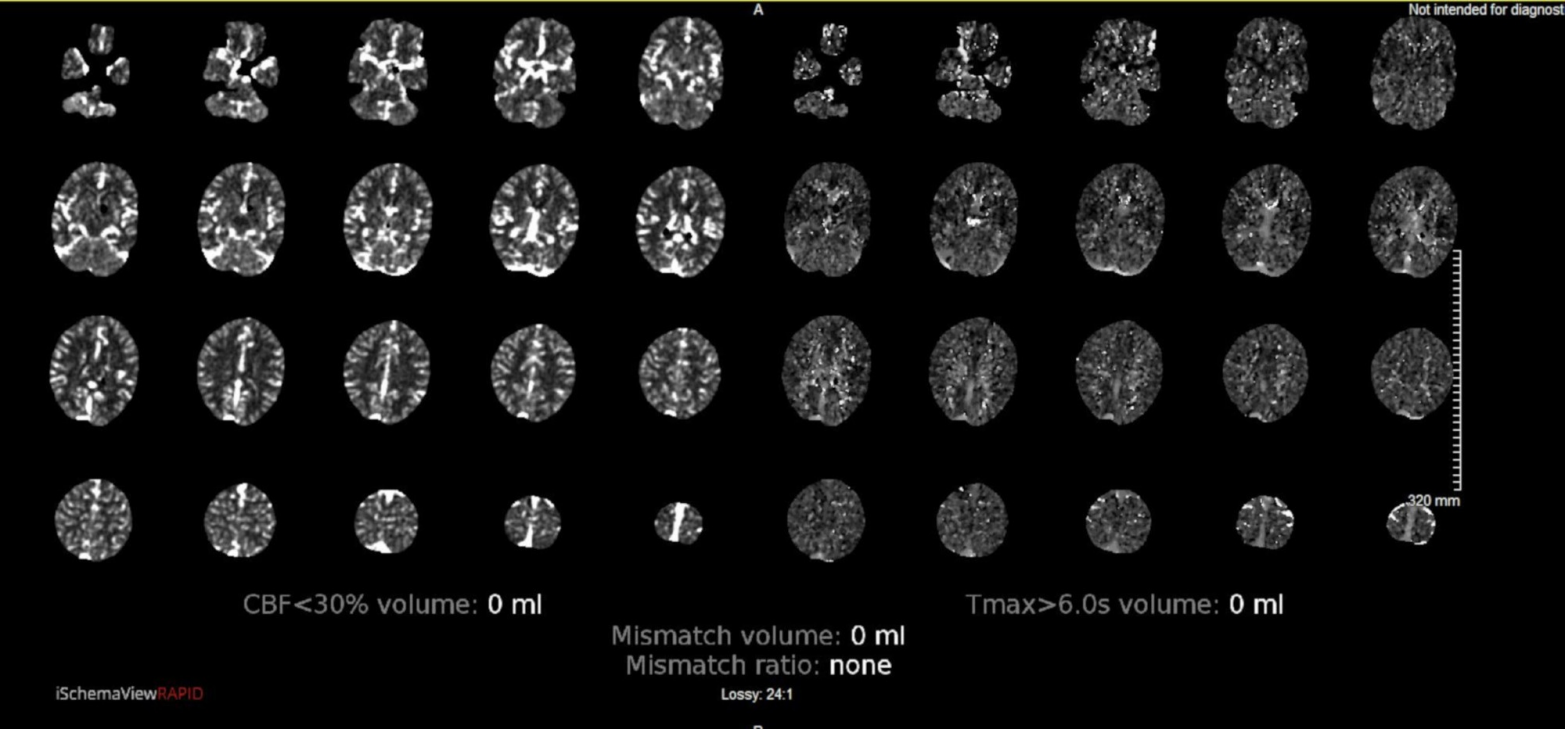
CT scan of the brain viewed with iSchemaView RAPID neuroimaging software revealed no areas of infarct. CT, computed tomography

**Figure 2:**
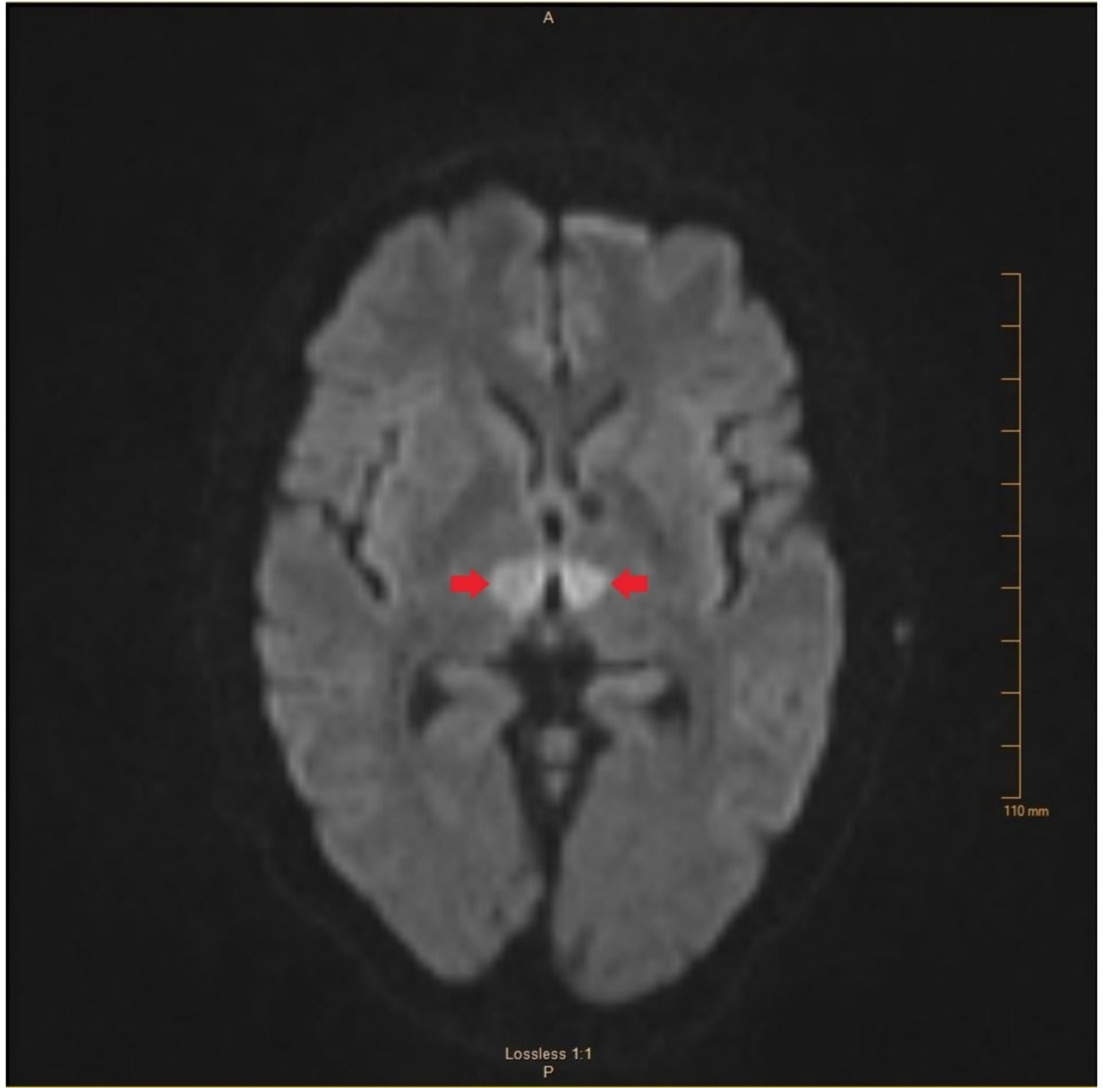
Diffusion-weighted MRI of the brain demonstrating increased signal in the distribution of the AOP. MRI, magnetic resonance imaging; AOP, artery of Percheron.

**Figure 3:**
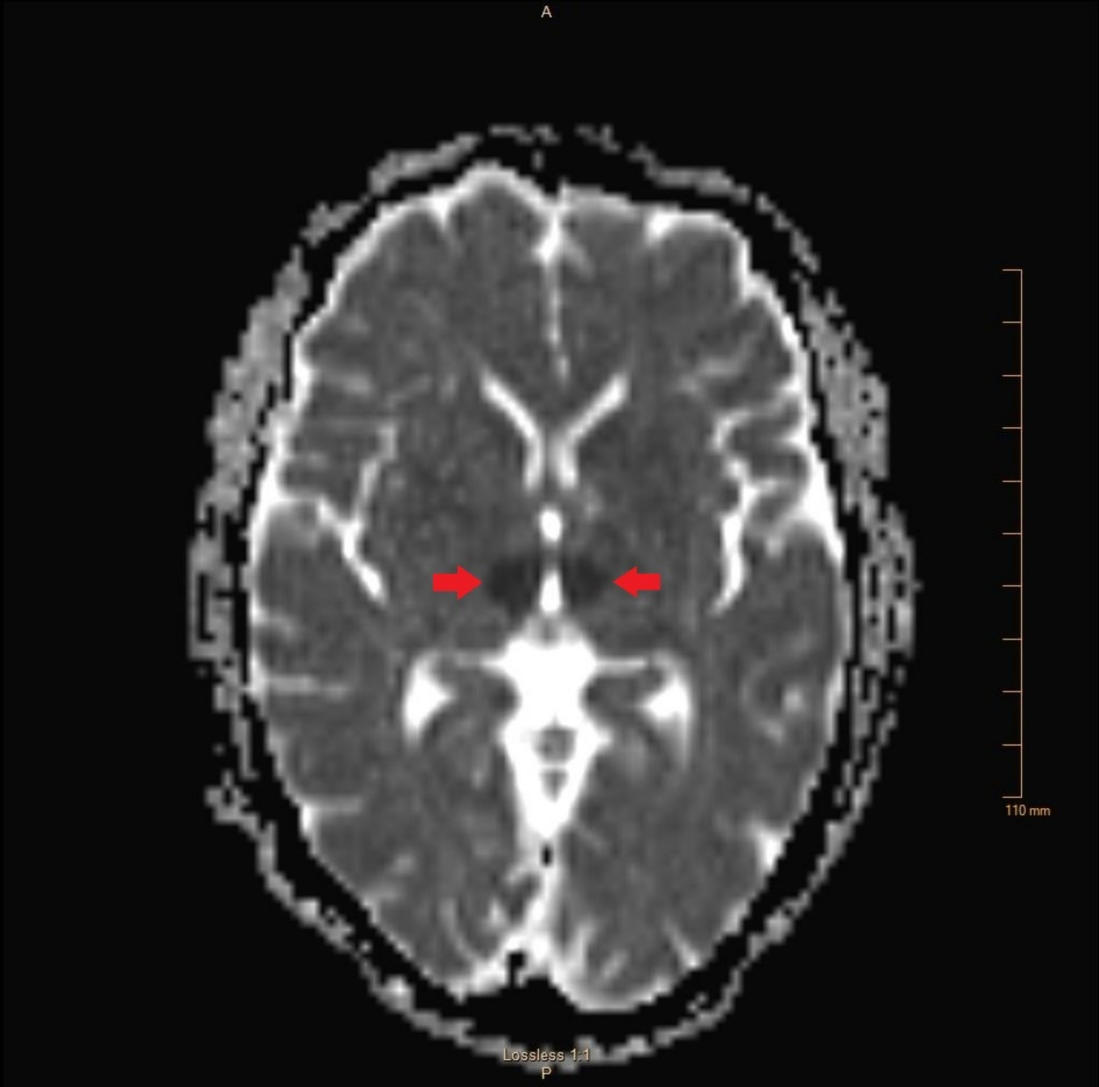
MRI with apparent diffusion coefficient confirming infarct in the area supplied by the AOP. MRI, magnetic resonance imaging; AOP, artery of Percheron.

Remaining history and review of systems were limited by the patient’s condition. Physical exam on arrival to the emergency department revealed a stuporous, nonverbal patient who was unresponsive to verbal stimuli. He had minimally reactive, unequal pupils, with the right measuring 4 mm and the left measuring 1 mm. He moved all his extremities in response to painful stimuli. A computed tomography (CT) scan of the head without contrast revealed no acute intracranial pathology. CT perfusion images, CT angiography, and iSchemaView RAPID neuroimaging technology showed no evidence of large vessel occlusion (Figure 1). Based on his vague presentation, the paucity of focal findings, and the lack of evidence of ischemia on imaging studies, there was very low suspicion for acute stroke at that time and he was, therefore, not a candidate for tissue plasminogen activator (tPA). Seven hours after his last witnessed normal baseline, diffusion-weighted magnetic resonance imaging (MRI) revealed acute infarcts in the bilateral thalami extending toward the ventral midbrain (Figures 2-3).

As part of the routine stroke work up, an echocardiogram showed evidence of left to right shunting suggestive of a patent foramen ovale, which was closed during his hospitalization. His clinical picture gradually improved over the course of his hospital stay, but he remained with several neurological deficits. By hospital day 22, he was awake and alert, and had recovered speaking and swallowing functions. However, he continued to suffer from recurrent falls, increased impulsivity, and impairments in cognition and memory.

## Discussion

This patient’s presentation is one of several that have been described in the literature for AOP infarcts. An occlusion of this artery may affect various thalamic nuclei with or without involvement of the rostral midbrain, resulting in several possible clinical pictures [10-11]. The degree of variation in thalamic vascular anatomy in conjunction with the wide spectrum of functions mediated by the thalamus help explain the variety in symptomatology associated with insults in this area and reinforce the need for awareness of the many ways AOP stroke can present.

The thalamus is a bilateral, egg-shaped, deep gray matter structure composed of several nuclei. It serves as a relay center for a broad array of neurological domains, including sensation, movement, arousal, cognition, behavior, and emotion. As such, insults affecting the thalamus have the potential to impact many different areas of function and cause a diverse set of deficits. The vascular supply of the thalamus includes four main arteries: the polar (tuberothalamic) artery, the inferolateral (thalamogeniculate) artery, the paramedian (thalamosubthalamic) artery, and the posterior choroidal arteries [2, 12]. There is considerable variation in the territory supplied by each artery. The exact supply area depends on the size of adjacent vascular territories, the presence or absence of certain inconstant arteries, and the anatomic differences in the four main arteries. Additionally, all of the thalamic arteries are terminal branches without functional anastomosis.

**Figure 4:**
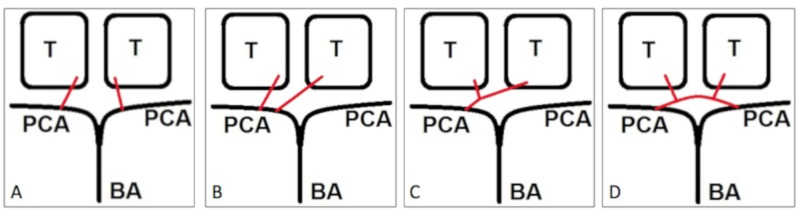
Paramedian thalamic artery variants (shown in red). A – Type I, normal anatomy; B – Type IIa, both paramedian arteries originate from the left P1 segment; C – Type IIb, the AOP originates unilaterally from the P1 segment and then bifurcates, supplying the bilateral paramedian thalamus and rostral midbrain; D – Type III, an arterial arcade connects the left and right P1 segments and gives rise to the paramedian arteries. T, thalamus; PCA, posterior cerebral artery; BA, basilar artery; AOP, artery of Percheron; P1, segment one of the posterior cerebral artery.

The AOP is an anatomic variant of the paramedian artery and arises from segment one of the posterior cerebral artery (P1). There are four major variants in the paramedian artery, first described in 1973 by Gerard Percheron, a French medical scientist [13]. Type I refers to normal anatomy and is characterized by symmetric paramedian arteries originating bilaterally from their corresponding P1 segments (Figure 4A). Type IIa has both paramedian arteries originating from either the left or right P1 artery (Figure 4B). Type IIb is the AOP, which originates from P1 unilaterally and then bifurcates to supply the bilateral paramedian thalamus and rostral midbrain (Figure 4C). This is the variant responsible for the bilateral thalamic infarct seen in this patient. Finally, Type III is characterized by an arterial arcade connecting the left and right P1 segments and giving rise to the paramedian arteries (Figure 4D).

The paramedian arteries typically supply the mediodorsal and intralaminar thalamic nuclei, which have diffuse inputs and outputs throughout the nervous system. The mediodorsal nucleus receives input from the amygdala, limbic basal ganglia, and olfactory cortex, and sends outputs to the frontal cortex [14]. The intralaminar nuclei receive information from the deep cerebellar nuclei, globus pallidus, and brainstem ascending reticular activating system (ARAS), and send projections to the striatum and cerebral cortex. The paramedian arteries can also supply portions of the upper brainstem, namely the interpeduncular nucleus, the decussation of the superior cerebellar peduncles, the medial aspect of the red nucleus, the third and fourth cranial nerve nuclei, and the anterior periaqueductal gray matter [1].

Patients with an AOP infarct present a diagnostic challenge due to the numerous functions relayed through the thalamus and the variation in its vascular anatomy. The classic features of a bilateral paramedian thalamic infarct are altered level of consciousness, vertical gaze palsy, and cognitive impairment [15]. However, the clinical features vary widely as reported in the literature, from mild symptoms such as dizziness and confusion to more severe signs like coma [3-4, 7-8]. The presentation may also include more classic signs of a stroke or focal lesion, as in the case of aphasia, dysarthria, and oculomotor disturbance [8]. Occasionally, patients present with more peculiar signs such as seizure or a Korsakoff-like psychosis [5-6]. Table 1 summarizes the common presentations seen in AOP infarction and their proposed anatomical correlates. This patient’s decreased level of arousal likely corresponded to damage to the intralaminar nuclei given its input from the brainstem ARAS, which mediates alert consciousness. His memory impairment could be attributed to infarction of the mediodorsal nucleus as it relays information from the amygdala. Memory loss may also arise from involvement of the anterior nucleus, which receives input from the hippocampus and mammillary bodies, and has been reported in a minority of AOP stroke cases [10-11].

**Table 1:** Common presentations seen in AOP infarction and their proposed anatomical correlates. AOP, artery of Percheron; ARAS, ascending reticular activating system; MLF, medial longitudinal fasciculus.

Presentation	Structures affected	Relevant projections
Coma	Intralaminar nuclei of the thalamus [14]	Brainstem ARAS
Memory impairment	Mediodorsal and anterior nuclei of the thalamus [5]	Amygdala, hippocampus, mammillary bodies
Behavioral changes	Mediodorsal and anterior nuclei of the thalamus [14]	Cingulate cortex, amygdala, limbic system, frontal lobes
Psychosis	Mediodorsal, intralaminar, and anterior nucleus of the thalamus [5-6]	Mammillary bodies, cingulate gyrus
Aphasia	Mediodorsal and intralaminar nuclei [16-18]	Frontal lobes, basal ganglia
Dysarthria	Mediodorsal nucleus of the thalamus [16-18]	Frontal lobes, basal ganglia, cerebellum
Oculomotor abnormalities (e.g. vertical gaze palsy)	Rostral midbrain (oculomotor nucleus, rostral interstitial nucleus of the MLF) [14]	Frontal eye fields
Cerebellar signs	Intralaminar nuclei of the thalamus [14]	Cerebellum

**Figure 5:**
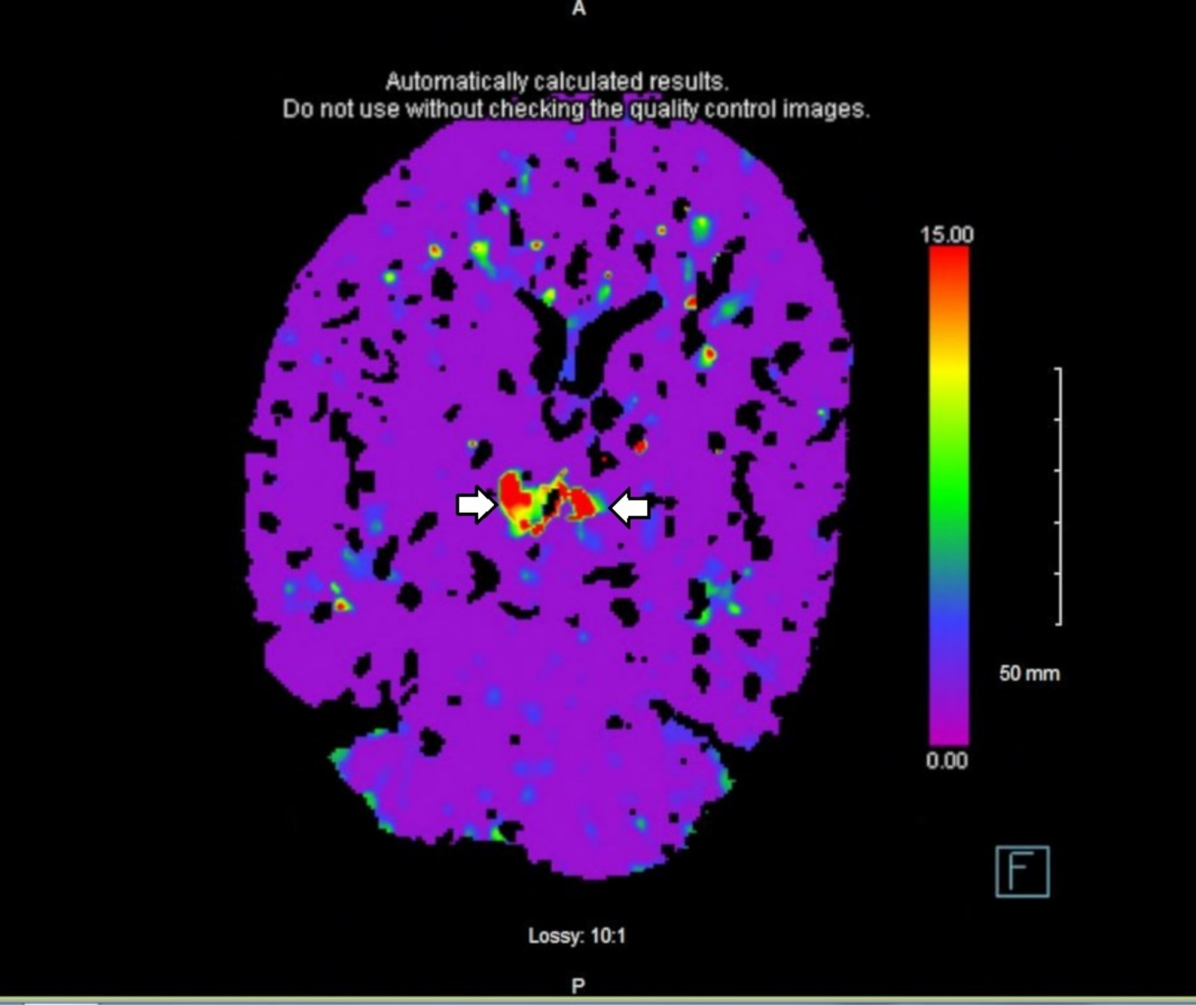
Time to drain CT perfusion source imaging showing decreased perfusion in the midbrain consistent with an AOP stroke. CT, computed tomography; AOP, artery of Percheron.

The CT imaging typically shows no abnormalities in acute AOP infarction. The CT angiography also tends to be normal, as occlusions in such small caliber arteries are usually not distinguishable. Likewise, CT perfusion imaging is useful in revealing large vessel occlusions, but often shows no filling defect in the case of very small vessels such as the AOP. iSchemaView RAPID stroke imaging software also failed to detect the AOP infarct in this patient (Figure 1). However, retrospective analysis of one of his CT perfusion sequences showed increased signal in the time to drain images (Figure 5). In contrast to CT imaging, MRI is usually diagnostic. A retrospective study of 18 cases of AOP stroke showed that 100% of cases were detected when MRI imaging was used, versus only 50% by CT scan [15]. Given the inability of many imaging modalities to resolve an AOP infarct, many patients with this type of stroke go undiagnosed for the first several hours until MRI is performed, as was the case in this patient. His vague presentation and negative CT scan of the brain were more suggestive of encephalopathy than a cerebrovascular event, for which reason MRI and other stroke workup was not initially done, resulting in delayed diagnosis and treatment. This case serves as a poignant reminder to consider AOP infarct and other thalamic pathology in the differential diagnosis when assessing patients with vague neurological deficits of unclear origin.

## Conclusions

Acute AOP infarcts continue to present a diagnostic challenge for clinicians in the acute setting owing to the diversity and inconsistency in presentation, frequent lack of localizing signs, and poor resolution on initial imaging. This has implications for treatment and prognosis, especially in settings offering tPA, where early detection and intervention significantly impacts functional outcomes for patients. It may not be cost effective to perform a complete stroke workup on all patients presenting with vague, atypical symptoms, but clinicians should keep thalamic pathology on the differential diagnosis given its involvement in many diverse neurological roles. Maintaining a high suspicion for thalamic infarct, with AOP occlusion as one etiology, and a low threshold for MRI in patients presenting acutely with otherwise unexplainable neurological symptoms may facilitate diagnosis and decrease morbidity in these patients.

## References

[1] Caruso P, Manganotti P, Moretti R (2016). Complex neurological symptoms in bilateral thalamic stroke due to Percheron artery occlusion. Vasc Health Risk Manag.

[2] Chen X, Wang Q, Wang X, Wong K (2017). Clinical features of thalamic stroke. Curr Treat Options Neurol.

[3] Hammersley D, Arora A, Dissanayake M, Sengupta N (2017). Fluctuating drowsiness following cardiac catheterisation: artery of Percheron ischaemic stroke causing bilateral thalamic infarcts. BMJ Case Rep.

[4] Bailey J, Khadjooi K (2016). Lesson of the month 1: artery of Percheron occlusion - an uncommon cause of coma in a middle-aged man. Clin Med.

[5] Kopelman MD (2018). What does a comparison of the alcoholic Korsakoff syndrome and thalamic infarction tell us about thalamic amnesia?. Neurosci Biobehav Rev.

[6] Zhou Y, Fox D, Anand A (2018). Artery of Percheron infarction as an unusual cause of Korsakoff’s syndrome. Case Rep Neurol Med.

[7] Turner J, Richardson T, Kane I, Vundavalli S (2014). Decreased consciousness: bilateral thalamic infarction and its relation to the artery of Percheron. Case Reports.

[8] Zappella N, Merceron S, Nifle C (2014). Artery of Percheron infarction as an unusual cause of coma: three cases and literature review. Neurocrit Care.

[9] Davous P, Bianco C, Duval-Lota AM, de Recondo J, Vedrenne C, Rondot P (1984). Aphasia caused by left paramedian thalamic infarction. Anatomo-clinical case. Rev Neurol.

[10] Arauz A, Patiño-Rodríguez HM, Vargas-González JC (2018). Clinical spectrum of artery of Percheron infarct: clinical-radiological correlations. J Stroke Cerebrovasc Dis.

[11] Lazzaro NA, Wright B, Castillo M (2018). Artery of percheron infarction: imaging patterns and clinical spectrum. Am J Neuroradiol.

[12] Castaigne P, Lhermitte F, Buge A, Escourolle R, Hauw J, Lyon-Caen O (2018). Paramedian thalamic and midbrain infarcts: clinical and neuropathological study. Ann Neurol.

[13] Percheron G (1973). The anatomy of the arterial supply of the human thalamus and its use for the interpretation of the thalamic vascular pathology. Z Neurol.

[14] Blumenfeld H (2010). Neuroanatomy Through Clinical Cases. Neuroanatomy Through Clinical Cases.

[15] Xu Z, Sun L, Duan Y, Zhang J, Zhang M, Cai X (2017). Assessment of Percheron infarction in images and clinical findings. J Neurol Sci.

[16] Ketteler D, Kastrau F, Vohn R, Huber Huber (2018). The subcortical role of language processing. High level linguistic features such as ambiguity-resolution and the human brain; an fMRI study. Neuroimage.

[17] Barbas H, García-Cabezas MÁ, Zikopoulos B (2013). Frontal-thalamic circuits associated with language. Brain Lang.

[18] Crosson B (2018). Thalamic mechanisms in language: a reconsideration based on recent findings and concepts. Brain Lang.

